# Association of Low Back Pain with Shift Work: A Meta-Analysis

**DOI:** 10.3390/ijerph20020918

**Published:** 2023-01-04

**Authors:** Ho-Ming Chen, Po-Yao Huang, Hung-Yi Chuang, Chao-Ling Wang, Chen-Cheng Yang, Peng-Ju Huang, Chi-Kung Ho

**Affiliations:** 1 Department of Occupational and Environmental Medicine, Kaohsiung Municipal Siaogang Hospital, Kaohsiung Medical University, Kaohsiung City 812, Taiwan; 2 Pharmacy Department, Kaohsiung Municipal Siaogang Hospital, Kaohsiung Medical University, Kaohsiung City 812, Taiwan; 3 Department of Occupational and Environmental Medicine, Kaohsiung Medical University Hospital, Kaohsiung Medical University, Kaohsiung City 807, Taiwan; 4 Department of Public Health and Environmental Medicine, and Research Center for Precision Environmental Medicine, Kaohsiung Medical University, Kaohsiung City 807, Taiwan; 5 Department of Orthopaedics, Kaohsiung Municipal Siaogang Hospital, Kaohsiung Medical University, Kaohsiung City 812, Taiwan

**Keywords:** shift work, low back pain, lumbago, occupational medicine, meta-analysis, health care worker

## Abstract

Shift work (SW) is the main working schedule worldwide, and it may cause sleep disorders, breast cancer, and cardiovascular disease. Low back pain (LBP) is a common problem in the workplace; however, the association between LBP and SW remains unclear. Therefore, we conducted a meta-analysis to determine the association between SW and LBP. This study was conducted in accordance with the Preferred Reporting Items for Systematic Reviews and Meta-Analyses guidelines. The PubMed, Embase, and Web of Science databases using a set of associated keywords were queried. The inclusion criteria were as follows: (1) adult employees hired by a company or organization; (2) SW exposure; and (3) the outcome of LBP according to examination or assessment. A total of 40 studies were included that met the inclusion criteria for the meta-analysis. SW was significantly associated with LBP (odds ratio [OR]: 1.31, 95% confidence interval [CI]: 1.18–1.47, *p* < 0.00001). Furthermore, it was observed that LBP was significantly associated with night shift (NS) (OR: 1.49, 95% CI: 1.24–1.82, *p* < 0.0001) but not with rotating shift (RS) (OR: 0.96, 95% CI: 0.76–1.22, *p* = 0.49). Moreover, LBP was significantly associated with SW in health care workers (HCWs) (OR: 1.40, 95% CI: 1.20–1.63, *p* < 0.0001) but not in non-HCWs (OR: 1.19, 95% CI: 0.94–1.50, *p* = 0.14). SW was significantly associated with LBP. Furthermore, the subgroup analysis showed that NS, but not RS, was associated with LBP. Compared with SW in non-HCWs, SW in HCWs was significantly associated with LBP.

## 1. Introduction

To date, shift work (SW) is an important issue in occupational medicine. Approximately 20% of the full-time workforce in Taiwan comprises shift workers [1]; the rate is around 30% in the United States [2], while it is 21% in Europe. According to the International Labour Organization, SW is defined as “a method of organization of working time in which workers succeed one another at the workplace.” Torquati et al. showed that SW increases the risk of poor mental health by 30% [3]. Furthermore, fatigue, insomnia, and various somatic diseases are common SW disorders [4].

Several cohort studies showed that SW at night was associated with the risk of coronary heart disease and incident atrial fibrillation [5], type 2 diabetes [5], ischemic stroke [6], breast cancer in females [7], non-alcoholic fatty liver disease [8], decreased brain functional connectivity [9], and obesity [10]. However, there was no significant association between SW and heart failure [11] or obstructive sleep apnea [12]. Additionally, quitting SW decreased coronary heart disease risk among women [13].

Low back pain (LBP) is a common disorder in humans. Globally, the age-standardized point prevalence of LBP in 21 regions was investigated and found to be around 5.6%, 13%, 9%, and 12% in Central Latin America, Australasia, Asia, and Europe, respectively, in 2017 [14]. Poor general health, physical and psychological stress, and characteristics of the person increase the risk of future episodes of LBP or sciatica [15].

Several studies have shown that SW is significantly associated with LBP. The rotating shift (RS), irregular shift, longer night shift (NS), and SW over 16 h were positively correlated to LBP [16,17]. However, NS over 16 h was associated with LBP, which was elevated when participants had sleep problems [16]. Many factors, such as SW, sleep disorders [18], poor mental health [3], and breast cancer [19], may cause LBP [17]. In contrast, other studies showed no significant association between SW and LBP [20,21]. Kawaguchi et al. showed no significant association between irregular SW and LBP (odds ratio [OR]: 1.1, 95% confidence interval [CI]: 0.6–1.9) [20]. A retrospective analysis using 13 years of occupational data from the National Longitudinal Survey of Youth, comprising approximately 11,000 American workers, showed no elevated risk of injury due to evening RS, night RS, or long-term working [21]. Given the inconsistent reports on the correlation between SW and LBP, this study aimed to investigate the relationship between SW and LBP.

## 2. Materials and Methods

### 2.1. Protocol and Registration

A systematic review and meta-analysis according to the Preferred Reporting Items for Systematic Reviews and Meta-Analyses (PRISMA) guidelines were conducted. This review protocol was registered at PROSPERO (registration number, CRD42022356707) and the Kaohsiung Medical University Hospital Institutional Review Board (KMUHIRB-EXEMPT(I)-20220009).

### 2.2. Data Sources and Search Terms

MEDLINE (PubMed), Embase, and Web of Science databases were queried on 1 September 2022, for related studies. There were no limitations to the publication dates and target keywords used to identify all articles. Two researchers (C-C Yang and H-M Chen) performed rudimentary searches using different keywords. The researchers separately proposed a set of key search words as follows for low back pain:

“Low Back Pain” [All Fields] OR “back pain low” [All Fields] OR (“Low Back Pain” [MeSH Terms] OR (“low” [All Fields] AND “back” [All Fields] AND “pain” [All Fields]) OR “Low Back Pain” [All Fields] OR (“back” [All Fields] AND “pains” [All Fields] AND “low” [All Fields])) OR “Low Back Pains” [All Fields] OR “pain low back” [All Fields] OR “pains low back” [All Fields] OR “Lumbago” [All Fields] OR “Lower Back Pain” [All Fields] OR “back pain lower” [All Fields] OR (“Low Back Pain” [MeSH Terms] OR (“low” [All Fields] AND “back” [All Fields] AND “pain” [All Fields]) OR “Low Back Pain” [All Fields] OR (“back” [All Fields] AND “pains” [All Fields] AND “lower” [All Fields])) OR “Lower Back Pains” [All Fields] OR “pain lower back” [All Fields] OR “pains lower back” [All Fields] OR “Low Back Ache” [All Fields] OR “ache low back” [All Fields] OR (“Low Back Pain” [MeSH Terms] OR (“low” [All Fields] AND “back” [All Fields] AND “pain” [All Fields]) OR “Low Back Pain” [All Fields] OR (“aches” [All Fields] AND “low” [All Fields] AND “back” [All Fields])) OR (“Low Back Pain” [MeSH Terms] OR (“low” [All Fields] AND “back” [All Fields] AND “pain” [All Fields]) OR “Low Back Pain” [All Fields] OR (“back” [All Fields] AND “ache” [All Fields] AND “low” [All Fields])) OR (“Low Back Pain” [MeSH Terms] OR (“low” [All Fields] AND “back” [All Fields] AND “pain” [All Fields]) OR “Low Back Pain” [All Fields] OR (“back” [All Fields] AND “aches” [All Fields] AND “low” [All Fields])) OR (“Low Back Pain” [MeSH Terms] OR (“low” [All Fields] AND “back” [All Fields] AND “pain” [All Fields]) OR “Low Back Pain” [All Fields] OR (“low” [All Fields] AND “back” [All Fields] AND “aches” [All Fields])) OR “Low Backache” [All Fields] OR “backache low” [All Fields] OR (“Low Back Pain” [MeSH Terms] OR (“low” [All Fields] AND “back” [All Fields] AND “pain” [All Fields]) OR “Low Back Pain” [All Fields] OR (“backaches” [All Fields] AND “low” [All Fields])) OR (“Low Back Pain” [MeSH Terms] OR (“low” [All Fields] AND “back” [All Fields] AND “pain” [All Fields]) OR “Low Back Pain” [All Fields] OR (“low” [All Fields] AND “backaches” [All Fields])) OR “low back pain postural” [All Fields] OR “Postural Low Back Pain” [All Fields] OR (“Low Back Pain” [MeSH Terms] OR (“low” [All Fields] AND “back” [All Fields] AND “pain” [All Fields]) OR “Low Back Pain” [All Fields] OR (“low” [All Fields] AND “back” [All Fields] AND “pain” [All Fields] AND “posterior” [All Fields] AND “compartment” [All Fields])) OR “low back pain recurrent” [All Fields] OR “Recurrent Low Back Pain” [All Fields] OR “low back pain mechanical” [All Fields] OR “Mechanical Low Back Pain” [All Fields] OR (“Low Back Pain” [MeSH Terms] OR (“low” [All Fields] AND “back” [All Fields] AND “pain” [All Fields]) OR “Low Back Pain” [All Fields])) 

And the mesh term is as follows for shift work:

(“Shift Work” [All Fields] OR “Schedule Shift Work” [All Fields] OR “Schedules Shift Work” [All Fields] OR “Work Schedule Shift” [All Fields] OR “Night Shift Work” [All Fields] OR “Shift Work Night” [All Fields] OR “Rotating Shift Work” [All Fields] OR “Shift Work Rotating” [All Fields] OR “Evening Shift Work” [All Fields] OR “Evening Shift” [All Fields] OR ((“shift” [All Fields] OR “shifted” [All Fields] OR “shifting” [All Fields] OR “shiftings” [All Fields] OR “shifts” [All Fields]) AND (“work” [MeSH Terms] OR “work” [All Fields]) AND (“evening” [All Fields] OR “evenings” [All Fields])) OR “Shift Worker” [All Fields] OR “Work Shift” [All Fields] OR ((“shift” [All Fields] OR “shifted” [All Fields] OR “shifting” [All Fields] OR “shiftings” [All Fields] OR “shifts” [All Fields]) AND (“work” [MeSH Terms] OR “work” [All Fields] )) AND “Shift Work” [All Fields] OR “schedule shift work” [All Fields] OR “schedules shift work” [All Fields] OR “work schedule shift” [All Fields] OR “Night Shift Work” [All Fields] OR “shift work night” [All Fields] OR “Rotating Shift Work” [All Fields] OR “shift work rotating” [All Fields] OR “Evening Shift Work” [All Fields] OR “Evening Shift” [All Fields] OR ((“shift” [All Fields] OR “shifted” [All Fields] OR “shifting” [All Fields] OR “shiftings” [All Fields] OR “shifts” [All Fields]) AND (“work” [MeSH Terms] OR “work” [All Fields]) AND (“evening” [All Fields] OR “evenings” [All Fields])) OR “Shift Worker” [All Fields] OR “Work Shift” [All Fields] OR ((“shift” [All Fields] OR “shifted” [All Fields] OR “shifting” [All Fields] OR “shiftings” [All Fields] OR “shifts” [All Fields]) AND (“work” [MeSH Terms] OR “work” [All Fields])). 

Appropriate modified search methods were used for EMBASE and the Web of Science databases.

### 2.3. Eligibility Criteria

The inclusion criteria were as follows: (1) SW exposure; (2) LBP, based on questionnaire assessment, lumbar spine computed tomography, or magnetic resonance imaging examination.

### 2.4. Study Selection Process

In the first screening, three investigators (H–M Chen, H–Y Chuang, and C–C Yang) individually assessed the abstracts of the preliminary articles included. Subsequently, in the second screening, two investigators (H–M Chen and P–Y Huang) performed full-text screening to identify articles that met the eligibility criteria and exclude those that were not eligible. The disagreements between H–M Chen and P–Y Huang regarding the eligibility of a study were resolved by three researchers (C–C Yang, C–L Wang, and P–J Huang) following a comprehensive evaluation.

### 2.5. Data Collection

In each eligible study, information regarding the study characteristics, SW and LBP cases, and the association between SW and LBP was obtained. Several attempts were made to contact the relevant authors to provide details in the event that the information was missing or inaccurate.

### 2.6. Study Characteristics

The study data was recorded with respect to the following variables: the country where the study was conducted; publication year; sampling framework (clinical- or community-based); sample size; characteristics of participants; and the number of outcome events (i.e., the number of LBP events), as appropriate.

### 2.7. Shift Work

The definition of SW is “work beyond regular working daytime hours”, including evening shift, NS, fixed shift, on-call shift, or RS [22,23,24,25].

### 2.8. Low Back Pain

The classification of LBP was as follows: questionnaires for LBP assessment. LBP was defined based on the individual study criteria.

### 2.9. Statistical Analysis

A calculation of the overall pooled prevalence ORs for LBP was made according to SW and non-SW exposures. A standard error (SE) of 95% CI was used for the OR. In this meta-analysis, the prevalence of OR and SE was reported. The main prevalence ORs and SEs were combined using a random-effects model meta-analysis to calculate the pooled prevalence OR and 95% CI for the primary outcome. A random-effects model was used to assess the possibility of heterogeneity regarding whether the ORs of the included studies originated from their characteristics [26], while I^2^ was used to report the heterogeneity among the enrolled studies. Moreover, separate subgroup meta-analyses for shift styles (NS and RS) and worker types (health care worker (HCW) and non-HCW) were performed. The Review Manager version 5.4 and R version 3.6.2 were used for all statistical analyses.

## 3. Results

### 3.1. Selected Studies

**Figure 1:**
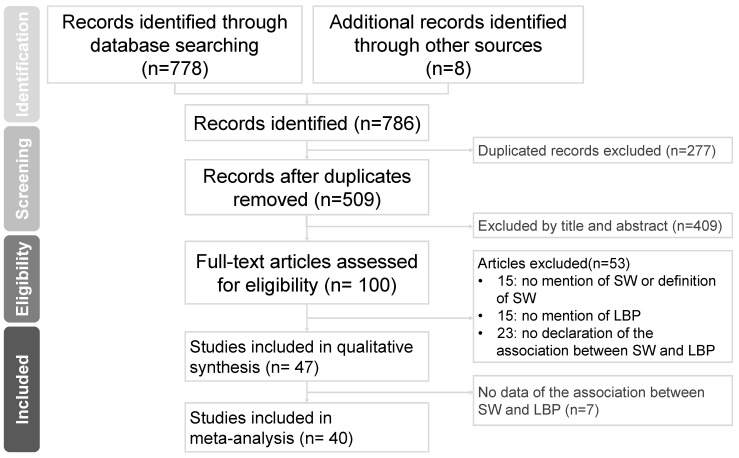
Preferred Reporting Items for Systematic Reviews and Meta-Analyses (PRISMA) flow diagram. SW, shift work; LBP, low back pain.

A summary of the literature search procedure is shown in Figure 1. Three different databases (PubMed, EMBASE, and Web of Science) were used, and additional records were identified through other sources, resulting in a total of 786 articles. In the next step, 277 articles were excluded owing to duplication; therefore, a total of 509 studies were screened for abstracts and titles. In the first phase of the screening process, 409 studies were excluded, leaving 100 for full-text screening. In the second phase of full-text screening, 53 studies did not meet the inclusion criteria owing to inappropriate study design, insufficient patient groups, and inappropriate patient group studies. Furthermore, 7 studies did not have adequate data to meet the quantitative synthesis criteria. Finally, we included 40 studies with 1,839,258 participants for meta-analysis [17,27,28,29,30,31,32,33,34,35,36,37,38,39,40,41,42,43,44,45,46,47,48,49,50,51,52,53,54,55,56,57,58,59,60,61,62,63,64,65].

### 3.2. Study Characteristics

Table 1 presents the 40 studies [17,27,28,29,30,31,32,33,34,35,36,37,38,39,40,41,42,43,44,45,46,47,48,49,50,51,52,53,54,55,56,57,58,59,60,61,62,63,64,65] that met our inclusion criteria; 31 studies used cross-sectional study designs [17,27,28,29,30,31,32,35,36,37,39,40,41,44,45,46,47,48,49,50,52,56,57,58,59,60,61,62,63,64,65]. Additionally, out of the remaining 9 studies, one study applied a prospective design [55], and 8 = eight used case-control study designs [33,34,38,42,43,51,53,54]. In 35 studies, the assessment of LBP was questionnaire-based [17,27,28,29,30,31,32,35,36,37,38,39,40,41,42,45,46,47,48,49,50,51,52,53,55,56,57,58,59,60,61,62,63,64,65], while in 3 studies [34,43,44], the assessment was based on a clinical diagnosis of LBP. Moreover, one study used the Occupational Prevention and Protection Service database, consisting of all safety reports and human resources information [33], and another used self-administrated instruments [54].

### 3.3. Results of Individual Studies

Table 1 shows the reported measures of the association between SW exposure and LBP. A total of thirteen studies reported a significant association between SW exposure and LBP [27,29,30,31,34,37,44,45,56,58,59,63,64]. However, the remaining two studies revealed a negative association of SW exposure with LBP [47,60].

### 3.4. Meta-Analysis

**Figure 2:**
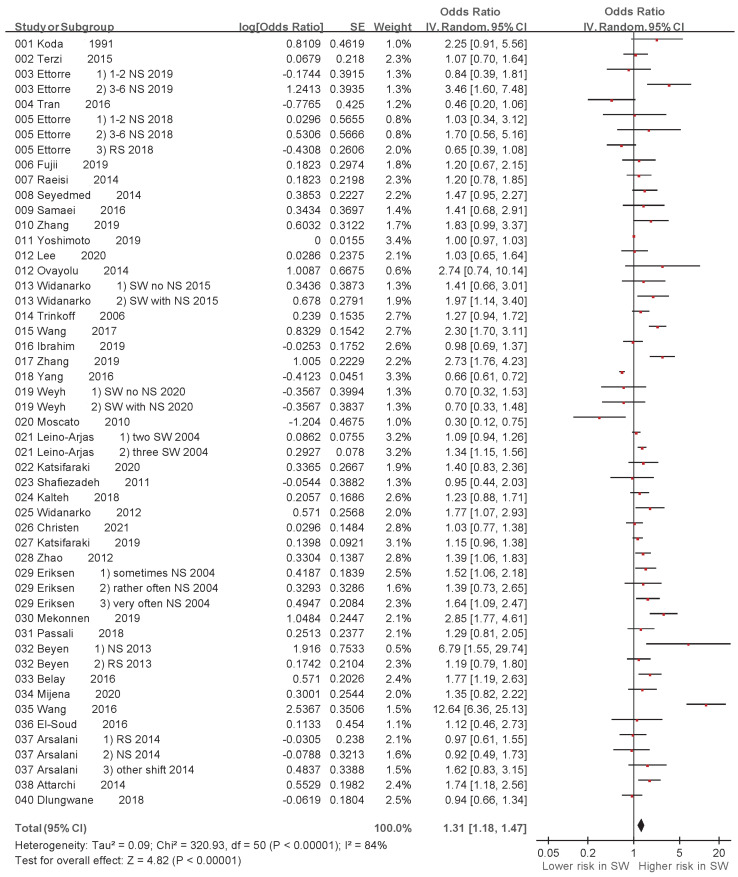
Shift work and odds ratio of low back pain in the 40 studies: a random-effects model (CI, confidence interval; SE, standard error) [17,27,28,29,30,31,32,33,34,35,36,37,38,39,40,41,42,43,44,45,46,47,48,49,50,51,52,53,54,55,56,57,58,59,60,61,62,63,64,65].

A random-effects model meta-analysis revealed variations in the association between exposure to SW and LBP (ORs derived from 40 studies) [17,27,28,29,30,31,32,33,34,35,36,37,38,39,40,41,42,43,44,45,46,47,48,49,50,51,52,53,54,55,56,57,58,59,60,61,62,63,64,65] (Table 1, Figure 2). The pooled prevalence OR was significant. The random-effects model meta-analysis indicated a significant positive association between SW and LBP (OR: 1.31, 95% CI: 1.18–1.47, *p* < 0.00001).

**Figure 3:**
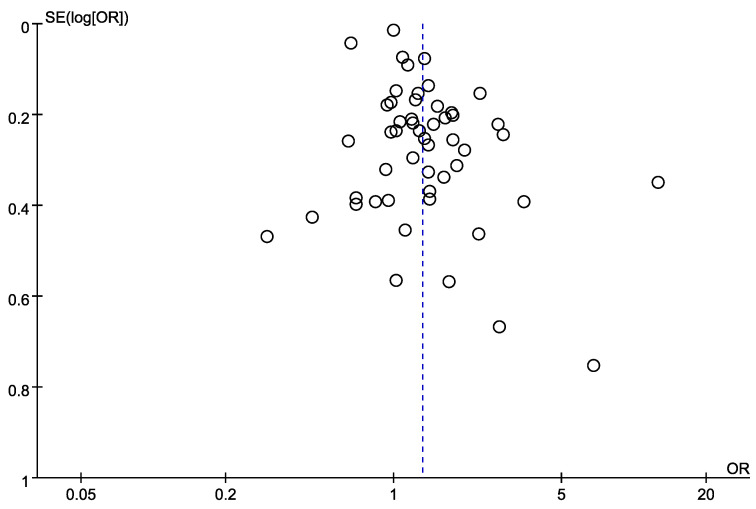
Funnel plot of log-transformed odds ratios of low back pain associated with shift work and standard errors for the 40 studies [17,27,28,29,30,31,32,33,34,35,36,37,38,39,40,41,42,43,44,45,46,47,48,49,50,51,52,53,54,55,56,57,58,59,60,61,62,63,64,65].

A funnel plot of the log-transformed ORs of the association of LBP with exposure to SW as well as the SEs of the 51 ORs showed that an adequate number of studies had small SEs (i.e., larger sample sizes) and smaller ORs (Figure 3).

### 3.5. Subgroup Analysis

**Figure 4:**
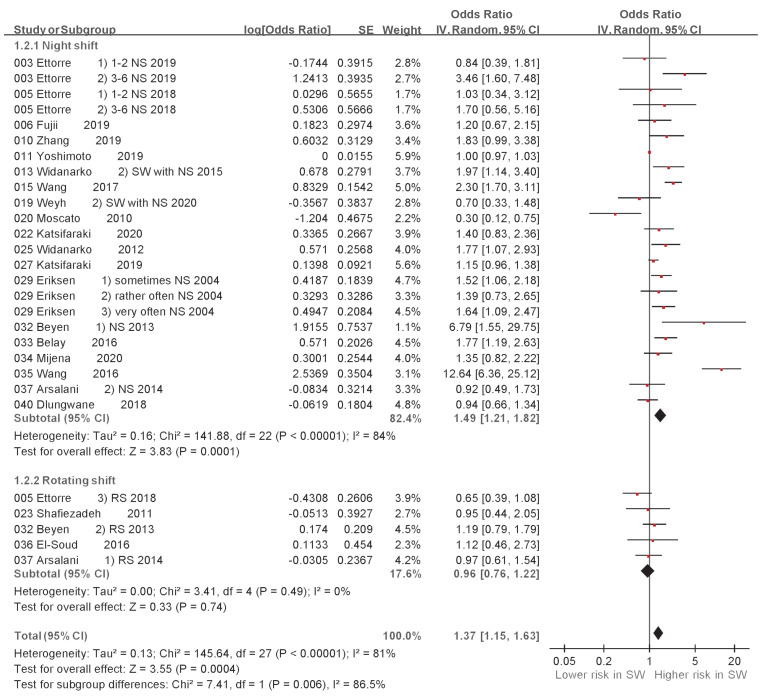
Subgroup analysis of odds ratio of low back pain based on night shift or rotating shift. CI, confidence interval; SE, standard error [27,28,30,31,33,34,35,36,37,38,40,41,46,47,51,56,57,58,59,61,62].

**Figure 5:**
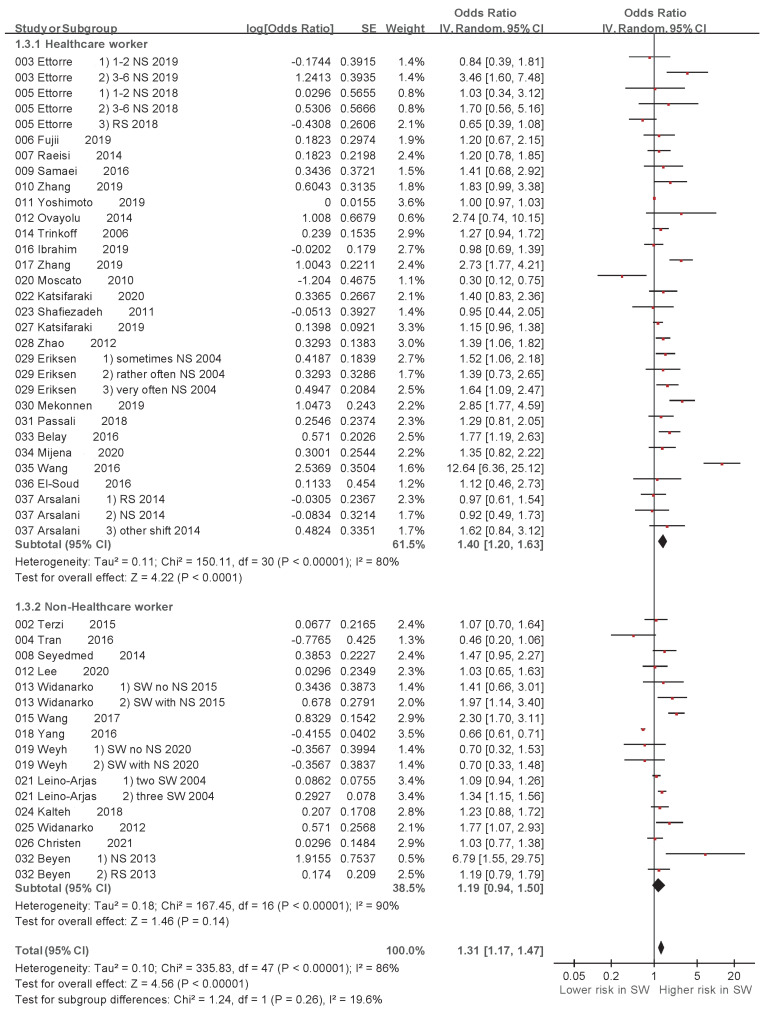
Subgroup analysis of odds ratio of low back pain associated with shift work based on health care worker (HCW) and non-HCW. CI, confidence interval; SE, standard error [17,27,28,30,31,32,33,34,36,37,38,39,40,41,43,44,45,46,47,48,49,50,51,52,53,54,55,56,57,58,59,60,61,62,63,64,65].

A random-effects model meta-analysis revealed variations in the association between exposure to different shift styles and LBP. The associations between NS and LBP (ORs derived from 19 studies) [27,28,30,31,33,34,35,37,38,40,41,46,47,56,57,58,59,61,63] and between RS and LBP (ORs derived from 5 studies) [28,31,33,36,52] are shown in Table 2 and Figure 4. The random-effects model meta-analysis indicated a significant association between NS and LBP (OR: 1.49, 95% CI: 1.21–1.82, *p* = 0.0001), while no significant association was found between RS and LBP (OR: 0.96, 95% CI: 0.76–1.22, *p* = 0.74). Furthermore, variations in the association between exposure to SW and LBP in HCWs and non-HCWs were revealed. The associations between SW and LBP in HCWs (ORs derived from 24 studies) [17,27,28,30,33,34,36,37,38,40,41,45,46,47,48,49,50,52,55,61,62,63,64,65] and between SW and LBP in non-HCWs (ORs derived from 13 studies) [31,32,39,43,44,51,53,54,56,57,58,59,60] are shown in Table 3 and Figure 5. The random-effects model meta-analysis indicated a significant association between SW and LBP in HCWs (OR: 1.40, 95% CI: 1.20–1.63, *p* < 0.00001), while no significant association was found between SW and LBP in non-HCWs (OR: 1.19, 95% CI: 0.94–1.50, *p* = 0.14).

## 4. Discussion

This study is a meta-analysis based on the original studies. In the meta-analysis of the 40 original studies, we found that SW was significantly associated with LBP (OR: 1.31, 95% CI: 1.18–1.47, *p* < 0.00001). Another meta-analysis conducted by Gohar et al. showed a statistically significant association between nursing in SW and sickness absence between 1990 and 2019 (OR: 1.47, 95% CI: 1.23–1.77, *p* < 0.01) [66]. Sun et al. showed that non-specific chronic LBP was significantly associated with working NSs in nurses [67]. Further, Jegnie et al. showed that working hours and SW had a statistically significant association with LBP in Ethiopia [68]. On the contrary, Moscato et al. [47] and Yang et al. [60] showed no significant association between SW and LBP. It was observed that the population in the former one had lower body mass index and the latter one obese worker only take 26.9% in total worker much less than the average in their country based on the journal named Our World in Data [69]. Hence, obesity may be probably one of the risk factors of SW. However, how the body weight influence on the association between SW and LBP requires further investigation.

In addition, although we know that different job descriptions and surroundings lead to different risks of LBP [59,70], the focus of the study was on SW styles without restrictions on occupation and area. In our meta-analysis, some original studies showed a significant association [27,29,30,31,34,37,44,45,56,58,59,63,64], whereas some studies revealed no significant association between SW and LBP. The reason for this difference may be the different study designs, study populations, and careers. Moreover, we found that some studies used different definitions for shift style, such as NS only, three-shift system, or occasionally SW. Similar to the study by Arsalani et al. [28], who categorized SW into morning shift, circulatory shift, NS, and others in their cross-sectional study of the Asian population. Beyen et al. used day shift, NS, and both as SW in a case-control study conducted in Europe [31]. Further, El-Soud et al. categorized SW into day shift and RS in a longitudinal study. The definition of SW seemed to vary between the West and East. Indeed, different work styles are required depending on the type of work. In this meta-analysis, we applied a broad definition of SW. It is difficult to collect information, which sometimes leads to less data, and a non-significant result is expected. Although a different study would have led to difficulty in collecting data, we still selected the most related data for analysis. In contrast, LBP results were not objective if only the questionnaire was used without professional identification. Work-related LBP [33], chronic LBP [63], and others have been reported. A more rigorous evaluation is needed in the future to understand the timing of SW that leads to LBP. Then, the result will be more convincing and allow employers to pay attention to the SW issue.

SW can cause many sleep problems, including reduced sleep quality, insomnia, and reduced sleep duration [71]. A previous study showed disruption of the circadian clock, especially due to NS and RS, leading to changes in melatonin and cortisol levels [72]. Morris et al. studied tissue physiology concerning circadian rhythms in the intervertebral disc and showed that changes in circadian rhythms cause harm to the intervertebral disc. [73]. In a mouse model, Dudek et al. showed that circadian rhythm disruptions lead to degenerative intervertebral disc disease [74]. These animal studies imply that SW leads to sleep interruption, which may cause circadian rhythm disruptions and LBP.

In the subgroup analysis, we found that LBP was significantly associated with NS (OR: 1.49, 95% CI: 1.24–1.82) but not with RS (OR: 0.96, 95% CI: 0.76–1.22). The exposure to NS may cause an increase in the body mass index (BMI) [75], which may be associated with LBP. However, Grundy et al. revealed that both permanent evening shift/NS and RS cause obesity [10]. Häuser et al. proved that increased BMI is associated with chronic LBP (OR: 1.09, 95% CI: 1.06–1.12, *p* < 0.0001) [10,76].

In some studies, we found that NS and RS led to health problems. According to a review by Feskanich et al., long-term NS increased the risk of hip and wrist fractures (OR: 1.37, 95% CI: 1.04–1.80) [77]. Additionally, Bukowska-Damska et al. revealed that NS workers had a lower mineral density of lumbar vertebral bones [78], while Quevedo et al. revealed that RS workers had a lower mineral density of lumbar vertebral bones [79]. According to these two studies, NS may be associated with a risk of fracture and low bone density, which may lead to LBP. However, how these potential confounding factors influence the association between SW and LBP require more study in the future.

In the subgroup analysis, we found that LBP was significantly associated with HCWs (OR: 1.40, 95% CI: 1.20–1.63). Stereotypically, HCWs are assumed to have more health information and knowledge, and fewer health problems than individuals in other occupations. However, a study by Kyle et al. study revealed that 69% (95% CI: 64.6–73.6) of Scottish nurses had obesity problems, especially in nurse groups, and non-health-related occupations (68.9%, 95% CI: 68.1–69.7) [80]. The reason for this result may be that HCWs need to shift patients, resulting in bend postures [81].

In this study, a positive relationship between SW and LBP is shown, resulting in many possible adverse health effects of SW, such as sleep disorder [18], poor mental health [3], and breast cancer [19]. The government and business organizations need to realize their responsibility concerning ways to decrease the occupational injury. Although some workers’ tasks may be demanding, employers can modify the task content as well as provide reasonable break time and regular health check-ups to develop a comfortable environment for the employee. Healthy employees would create more worker power and decrease the burden of social welfare.

## 5. Conclusions

In conclusion, SW was significantly associated with LBP according to the meta-analysis of 40 studies. Compared with non-SW, NS showed a significant association with LBP, while RS was not significantly associated with LBP. Furthermore, HCWs showed a significant association with LBP. The possible mechanisms require further investigation.

## Figures and Tables

**Table 1:** Characteristics of studies included in the meta-analysis (N = 40).

First Author (Year/Journal), Country	Study Design	Participants (Number)	Population	Sex	Exposure Variable	Outcome Measures	Number of Outcome Events/Cases	Comparison	OR (95% CI)	Source
Koda (1991) [42], Japan	Case-control study	Clinical nurse (947) and clerical worker (300)	Community population in Japan	Women only	SW (three shifts)	LBP	SW: 129; non-SW: 142	Questionnaire: LBP yes/no	2.25 (0.91–2.59)	Table 5 p. 416
Terzi (2015) [53], Turkey	Case-control study	Supermarket employee (365)	Community population in Çankaya, Turkey	Men and women combined	SW	LBP	SW: 87; non-SW: 135	Questionnaire: LBP yes/no	1.07 (0.6981–1.6407)	Table 2 calculation
Ettorre (2019) [34], Italy	Case-control study	Female nurse (1530)	Female rotating shift registered nurses	Women only	NS	WRLBP	Number of NS: 0: 10 1–2: 84 3–6: 110	Clinical diagnosis LBP	1–2 NS: 0.84 (0.39–1.81) 3–6 NS: 3.46 (1.6–7.47)	Table 2 p. 485
Tran (2016) [54], Vietnam	Case-control study	Female workers of the industrial zone in Vietnam	Female worker of 10 factories in Vietnam	Women only	SW	After-shift MSD syndrome in low back	SW: 95; non-SW: 23	Self-administrated instruments	0.46 (0.2–1.1)	Table 2 p. 742
Ettorre (2018) [33], Italy	Case-control study	Female rotating shift-registered nurse (671)	Female rotating shift-registered nurse in Italy	Women only	NS number in 7 days; RS	WRALBP	Number of NS 0: 5 1–2: 42 3–6: 46 RS: 31 Control: 81	Occupational Prevention and Protection Service database for safe reports and Human Resources information	1–2 NS: 1.03 (0.34–3.1) 3–6 NS: 1.7 (0.56–5.18) RS: 0.65 (0.39–1.1)	Table 2 p. 55, Table 5 p. 57
Fujii (2019) [38], Japan	Case-control study	Female nurse (3066)	Female nurse from 12 hospitals in Japan	Women only	NS	LBP	NS: 155; non-SW: 33	Questionnaire: LBP yes/no	1.2 (0.67–2.13)	Table 2 p. 6
Raeisi (2014) [50], Iran	Cross-sectional study	Nursing personnel in a large general hospital in Tehran, Iran (650)	Nursing personnel with at least 1 year experience	Men and women combined	SW	Low back disorder	SW: 219; non-SW: 98	Questionnaire: LBP yes/no	1.2 (0.78–1.94)	Table 2 p. 161 calculation
Seyedmed (2014) [51], Iran	Case-control study	Industrial worker (511)	Rubber factory worker in 2011–2013 in the city of Yazd, Iran	Men and women combined	SW	LBP	SW: 239; non-SW: 52	General Health Questionnaire score	1.47 (0.95–2.26)	Table 2 p. 214
Samaei (2016) [17], Iran	Cross-sectional study	Randomly selected nursing personnel (243)	Randomly selected nursing personnel, Iran	Men and women combined	SW	LBP	SW: 145; non-SW: 24	NMQ	1.41 (0.68–2.91)	Table 2 p. 5 calculation
Zhang (2019) [63], China	Cross-sectional study	Sonographers (248)	Sonographers in General Hospital Guangdong province	Men and women combined	NS	LBP	NS: 118; non-SW: 24	Questionnaire: LBP yes/no	1.83 (0.99–3.37)	Table 5 calculation
Yoshimoto (2019) [61], Japan	Cross-sectional study	Nursing personnel (1075)	Nursing personnel in Kameda Medical Centre at Chinba, Japan	Men and women combined	NS	LBP	NR	Questionnaire: LBP yes/no	1.00 (0.97–1.04)	Table 2 p. 5
Ovayolu (2014) [48], Turkey	Cross-sectional study	Nurse in intensive care units (114)	Nurse in intensive care units in the province of Gaziantep, Turkey	Men and women combined	SW	LBP	SW: 34; non-SW: 62	Questionnaire: LBP + Visual Analogue Scale	2.74 (0.74–10.14)	Table 1 p. 72 calculation
Lee (2020) [43], China	Case-control study	Manual worker (406)	Workers in Henan, Hubei, Guangdong province, China	Men only	SW	LBP	SW: 59; non-SW: 81	Difference-in-difference analysis	1.03 (0.65–1.64)	Table 2 p. 5 calculation
Widanarko (2015) [59], Indonesian	Cross-sectional study	Indonesian coal mining workers (1565)	Coal mining workers, Indonesian	Men and woman combined	SW without NS; SW with NS	LBP	SW no NS: 13; SW with NS: 218; non-SW: 28	NMQ	SW without NS: 1.41 (0.66–3.02) SW with NS: 1.97 (1.14–3.43)	Table 3 p. 164
Trinkoff (2006) [55], US	Three-wave longitudinal survey	Registered nurse (2617)	Registered nurse, US	Men and women combined	SW	LBP	NR	NMQ	1.27 (0.94–1.72)	Table 2 p. 968
Wang (2017) [56], China	Cross-sectional study	Taxi-driver of three major taxi companies in Jinan, China (719)	Taxi-driver of three major taxi companies in Jinan, China	Men and women combined	NS	LBP	NR	NMQ	2.3 (1.7–3.2)	Table 2 p. 3
Ibrahim (2019) [65], Malaysia	Cross-sectional study	Nurse aged 25–60 years working for six public hospitals of Penang, Malaysia (1292)	Nurse aged 25–60 years working for six public hospitals, Malaysia	Men and women combined	SW	LBP	SW: 819; non-SW: 170	The BACKS Tool questionnaire (self-administered)	0.98 (0.69–1.37)	Table 5 p. 7 of 12 calculation
Zhang (2019) [63], China	Cross-sectional study	Ambulance workers including doctors, nurses, and drivers (1560)	Ambulance workers in 38 tertiary hospitals in Shandong, China	Men and women combined	SW	Chronic LBP	SW: 60; non-SW: 45	NMQ	2.73 (1.77–4.23)	Table 4 p. 6
Yang (2016) [60], US	Cross-sectional study	2010 National Health Interview Survey in US (13924)	Database, US	Men and women combined	SW	LBP	SW: 873; non-SW: 2175	NHIS core questionnaire	0.66 (0.61–0.72)	Table 3 p. 7 calculation
Weyh (2020) [57], German	Cross-sectional study	Welders from 34 companies in German steel industry (145)	Steel industrial welders, German	Men and women combined	SW no NS; SW with NS	LBP	SW no NS: 28; SW with NS: 27; non-SW: 46	NMQ	SW without NS: 0.7 (0.32–1.47) SW with NS: 0.7 (0.33–1.57)	Table 1 p. 5
Moscato (2010) [47], Italy	Cross-sectional study	Operating room nurse in nine hospitals in Room (225)	Operating room nurse in nine hospitals in Room, Italy	Men and women combined	NS diurnals/nocturnals	Intensity of LBP	NS: 64; Non-SW: 89	Questionnaire: LBP yes/no NMQ	0.3 (0.12–0.78)	Table 2 p. 457
Leino-Arjas (2004) [44], Finland	Cross-sectional study	Patients hospitalized due to severe lumbar intervertebral disc disorders from the Finland Hospital Discharge Register (1783616)	Database, Finland	Men and women combined	Two SW; three SW	LBP	NR	Diagnosis (ICD-10: M51.1–51.9)	2 SW: 1.09 (0.94–1.26) 3 SW: 1.34 (1.15–1.56)	Table 3 p. 519
Katsifaraki (2020) [41], Norway	Cross-sectional study	Nurse with three-shift rotation, Norway (679)	Nurse with three-shift rotation, Norway	Men and women combined	NS	LBP	NR	Questionnaire: LBP yes/no	1.4 (0.83–2.34)	Table 3 p. 6
Shafiezadeh (2011) [52], Iran	Cross-sectional study	Nurses in a large hospital in Ahwaz, southwestern Iran (195)	Nurses in a large hospital in Ahwaz, southwestern Iran	Men and women combined	RS	LBP	RS: 46; non-SW: 15	NMQ	0.95 (0.44–2.03)	Table 3 p. 162 calculation
Kalteh (2018) [39], Iran	Cross-sectional study	Employee on offshore oil and gas installations, South Iran (1157)	Employee on offshore oil and gas installations, Iran	Men and women combined	SW	LBP	SW: 61; non-SW: 207	NMQ	1.23 (0.88–1.71)	Table 3 p. 4 calculation
Widanarko (2012) [58], Indonesian	Cross-sectional study	Coal mining industry workers, Indonesian (1294)	Coal mining industry workers, Indonesian	Men and women combined	NS	LBP	NR	Questionnaire: LBP yes/no NMQ	1.77 (1.07–2.93)	p. 5735 right column, result section, line10
Christen (2021) [32], Norway	Cross-sectional study	Data from the Troms Study, Norway (2332)	Data from Troms Study, a longitudinal population-based cohort study, Norway	Men and women combined	SW	LBP	NR	Questionnaire: LBP yes/no	1.03 (0.77–1.37)	Table 2
Katsifaraki (2019) [40], Norway	Cross-sectional study	Shift work nurses working in public hospitals in Norway (679)	Public hospitals, Norway	Men and women combined	NS	LBP	NR	Questionnaire: pain site: low back	1.15 (0.96–1.38)	Table 1
Zhao (2012) [64], Australia, NZ, and UK	Cross-sectional study	Nurses (5280)	Nurses and Midwives’ e-cohort Study (NMeS)	Men and women combined	SW	LBP	SW: 174; non-SW: 145	NMQ	1.39 (1.06–1.83)	Table 1 calculation
Eriksen (2004) [37], Norway	Cross-sectional study	Randomly selected Norwegion nurses’ aides (4266)	Norwegion nurses’ aides, Norway	Men and women combined	NS: sometimes/ rather often/ very often	LBP	NR	Questionnaire: LBP yes/no	Sometimes:1.52 (1.06–2.19) Rather often: 1.39 (0.73–2.63) Very often: 1.64 (1.09–2.49)	Table 4 p. 402
Mekonnen (2019) [45], Ethiopia	Cross-sectional study	Nurses (422)	Selected by random sampling technique, Ethiopia	Men and women combined	SW	LBP	SW: 227; non-SW: 39	NMQ	2.85 (1.77–4.61)	Table 2
Passali (2018) [49], Greece	Cross-sectional study	Nurses (394)	Nurses in the capital of Greece	Men and women combined	SW	LBP	SW: 198; non-SW: 78	NMQ	1.29 (0.81–2.05)	Table 3 p. 4 calculation
Beyen (2013) [31], Ethiopia	Cross-sectional study	Primary, secondary, or higher institution (college or university) teacher in Gondar town, Ethiopia (662)	Primary, secondary, or higher institution (college or university) teacher in Gondar town, Ethiopia	Men and women combined	NS, RS	LBP	NS: 17; RS: 70; non-SW: 259	NMQ	NS: 6.79 (1.55- 29.74) RS: 1.19 (0.79- 1.80)	Table 6 calculation
Belay (2016) [30], Ethiopia	Cross-sectional study	Nurses working in public hospitals of Addis Ababa, Ethiopia (395)	Nurses working in public hospitals of Addis Ababa, Ethiopia	Men and women combined	NS	LBP	NS: 94; non-NS: 86	self-administered questionnaire	1.77 (1.19–2.65)	Table 6a
Mijena (2020) [46], Ethiopia	Cross-sectional study	Nurses working in public hospitals of Harari region and Dire Dawa city administration, Ethiopia (404)	Nurses working in public hospitals of Harari region and Dire Dawa city administration, Ethiopia	Men and women combined	NS	LBP	NS: 126; non-NS: 28	self-administered questionnaire	1.35 (0.82–2.25)	Table 6
Wang (2016) [27], China	Cross-sectional study	Nurses working in three class-A hospitals, China (909)	Nurses working in hospitals, China	Men and women combined	NS	LBP	NS: 582; non-SW: 25	self-administered questionnaire	12.64 (6.36–25.12)	Table 3 calculation
El-Soud (2016) [36], Egypt	Cross-sectional study	Nurse working in Zagazig University Hospitals, Egypt (150)	Nurse working in Zagazig University Hospitals, Egypt	Women only	RS	LBP	RS: 76; non-SW: 43	self-administered questionnaire	1.12 (0.46–2.7)	Table 5 p. 112
Arsalani (2014) [28], Iran	Cross-sectional study	Nurses working in public hospitals in Tehran, Iran (606)	Nurses working in public hospitals in Tehran, Iran	Men and women combined	RS; NS; other	LBP	DS: 39 RS: 111 NS: 26 Other: 28	self-administered questionnaire	RS: 0.97 (0.61–1.55) NS: 0.92 (0.49–1.73) Other: 1.62 (0.84–3.15)	Table 1 calculation
Attarchi (2014) [29], Iran	Cross-sectional study	Health care workers in Tehran, Iran (454)	Nurses working in public hospitals in Tehran, Iran	Men and women combined	SW	LBP	SW: 182; non-SW: 79	NMQ	1.74 (1.18–2.56)	Table 1 calculation
Dlungwane (2018) [35], South Africa	Cross-sectional study	Nurse at a regional hospital, South Africa (373)	Nurse at a regional hospital, South Africa	Men and women combined	NS	LBP	NR	self-administered questionnaire	0.94 (0.66–1.34)	Table 4

CI, confidence ratio; DS, day shift; LBP, low back pain; NHIS, National Health Interview Survey; NMQ, Nordic Musculoskeletal Questionnaire; NR, NS, night shift; OR, odds ratio; RS, rotating shift; SW, shift work; MSD, musculoskeletal disorders; WRALBP, work-related acute low back pain; WRLBP, work-related low back pain; US, United States.

**Table 2:** Subgroup analysis of odds ratios based on participants exposed to night shifts or rotating shifts.

Subgroup	Pooled Odds Ratio	95% CI
Study Participants		
** ** **Night shift**		
Ettorre (2019) [34], Italy, Number of NS 1–2	0.84	0.39–1.81
Ettorre (2019) [34], Italy, Number of NS 3–6	3.46	1.6–7.47
Ettorre (2018) [33], Italy, Number of NS 1–2	1.03	0.34–3.1
Ettorre (2018) [33], Italy, Number of NS 3–6	1.7	0.56–5.18
Fujii (2019) [38], Japan	1.2	0.67–2.13
Zhang (2020) [62], China	1.8280	0.9914–3.3704
Yoshimoto (2019) [61], Japan	1.00	0.97–1.04
Widanarko (2015) [59], Indonesian, SW with NS	1.97	1.14–3.43
Wang (2017) [56], China	2.3	1.7–3.2
Weyh (2020) [57], German, SW with NS	0.7	0.33–1.57
Moscato (2010) [47], Italy	0.3	0.12–0.78
Katsifaraki (2020) [40], Norway	1.4	0.83–2.34
Widanarko (2012) [58], Indonesian	1.77	1.07–2.93
Katsifaraki (2019) [40], Norway	1.15	0.96–1.38
Eriksen (2004) [37], Norway, sometimes	1.52	1.06–2.19
Eriksen (2004) [37], Norway, rather often	1.39	0.73–2.63
Eriksen (2004) [37], Norway, very often	1.64	1.09–2.49
Beyen (2013) [31], Ethiopia, NS	6.7934	1.5518–29.7393
Belay (2016) [30], Ethiopia	1.77	1.19–2.65
Mijena (2020) [46], Ethiopia	1.35	0.82–2.25
Wang (2016) [27], China	12.6377	6.3568–25.1247
Arsalani (2014) [28], Iran, NS	0.9242	0.4924–1.7349
Dlungwane (2018) [35], South Africa	0.94	0.66–1.34
** ** **Subtotal**	**1.49**	**1.21–1.82**
** ** **Rotating shift**		
57. Ettorre (2018) [33], Italy, RS	0.65	0.39–1.1
342. Shafiezadeh (2011) [52], Iran	0.9471	0.4425–2.0270
504. Beyen (2013) [31], Ethiopia, RS	1.1903	0.7881–1.7978
508. El-Soud (2016) [36], Egypt	1.12	0.46–2.7
509. Arsalani (2014) [28], Iran, RS	0.9700	0.6084–1.5465
**Subtotal**	**0.96**	**0.76–1.22**

CI, confidence interval; NS, night shift; RS, rotating shift.

**Table 3:** Subgroup analysis of odds ratio for healthcare workers (HCWs) and non-HCWs exposed to shift work.

Subgroup	Pooled Odds Ratio	95% CI
Study Participants		
** ** **Healthcare worker**		
38. Ettorre (2019) [34], Italy, number of NS 1–2	0.84	0.39–1.81
38. Ettorre (2019) [34], Italy, number of NS 3–6	3.46	1.6–7.47
57. Ettorre (2018) [33], Italy, number of NS 1–2	1.03	0.34–3.1
57. Ettorre (2018) [33], Italy, number of NS 3–6	1.7	0.56–5.18
57. Ettorre (2018) [33], Italy, RS	0.65	0.39–1.1
67. Fujii (2019) [38], Japan	1.2	0.67–2.13
110. Raeisi (2014) [50], Iran	1.2	0.78–1.94
160. Samaei (2016) [17], Iran	1.4097	0.6831–2.9094
176. Zhang (2019) [62], China	1.8280	0.9914–3.3704
189. Yoshimoto (2019) [61], Japan	1.00	0.97–1.04
195. Ovayolu (2014) [48], Turkey	2.7419	0.7411–10.1444
241. Trinkoff (2006) [55], US	1.27	0.94–1.72
248. Ibrahim (2019) [65], Malaysia	0.9750	0.6917–1.3743
250. Zhang (2019) [63], China	2.732	1.765–4.229
278. Moscato (2010) [47], Italy	0.3	0.12–0.78
312. Katsifaraki (2020) [41], Norway	1.4	0.83–2.34
342. Shafiezadeh (2011) [52], Iran	0.9471	0.4425–2.0270
406. Katsifaraki (2019) [40], Norway	1.15	0.96–1.38
438. Zhao (2012) [64], Australia, NZ, and UK	1.3915	1.0602–1.8263
481. Eriksen (2004) [37], Norway, sometimes	1.52	1.06–2.19
481. Eriksen (2004) [37], Norway, rather often	1.39	0.73–2.63
481. Eriksen (2004) [37], Norway, very often	1.64	1.09–2.49
493. Mekonnen (2019) [45], Ethiopia	2.853	1.766–4.609
496. Passali (2018) [49], Greece	1.2857	0.8069–2.0487
505. Belay (2016) [30], Ethiopia	1.77	1.19–2.65
506. Mijena (2020) [46], Ethiopia	1.35	0.82–2.25
507. Wang (2016) [27], China	12.6377	6.3568–25.1247
508. El-Soud (2016) [36], Egypt	1.12	0.46–2.7
509. Arsalani (2014) [28], Iran, RS	0.9700	0.6084–1.5465
509. Arsalani (2014) [28], Iran, NS	0.9242	0.4924–1.7349
509. Arsalani (2014) [28], Iran, other shift	1.62	0.84–3.15
** ** **Subtotal**	**1.40**	**1.20–1.63**
** ** **Non-healthcare worker**		
12. Terzi (2015) [53], Turkey	1.0702	0.6981–1.6407
41. Tran (2016) [54], Vietnam	0.46	0.2–1.1
150. Seyedmed (2014) [51], Iran	1.47	0.95–2.26
218. Lee (2020) [43], China	1.0290	0.6460–1.6390
232. Widanarko (2015) [59], Indonesian, SW no NS	1.41	0.66–3.02
232. Widanarko (2015) [59], Indonesian, SW with NS	1.97	1.14–3.43
247. Wang (2017) [56], China	2.3	1.7–3.2
253. Yang (2016) [60], US	0.6621	0.6061–0.7233
276. Weyh (2020) [57], German, SW no NS	0.7	0.32–1.47
276. Weyh (2020) [57], German, SW with NS	0.7	0.33–1.57
300. Leino-Arjas (2004) [44], Finland, two SW	1.09	0.94–1.26
300. Leino-Arjas (2004) [44], Finland, three SW	1.34	1.15–1.56
344. Kalteh (2018) [39], Iran	1.2284	0.8827–1.7095
367. Widanarko (2012) [58], Indonesian	1.77	1.07–2.93
399. Christen (2021) [32], Norway	1.03	0.77–1.37
504. Beyen (2013) [31], Ethiopia, NS	6.7934	1.5518–29.7393
504. Beyen (2013) [31], Ethiopia, RS	1.1903	0.7881–1.7978
** ** **Subtotal**	**1.09**	**0.94–1.50**

CI, confidence interval; NS, night shift; RS, rotating shift; SW, shift work; UK, United Kingdom; NZ, New Zealand.

## Data Availability

The original contributions presented in the study are included in the article; further inquiries can be directed to the corresponding author.
